# The Transformation of The Indian Healthcare System

**DOI:** 10.7759/cureus.39079

**Published:** 2023-05-16

**Authors:** Ankit Kumar

**Affiliations:** 1 Respiratory Medicine, King George's Medical University, Lucknow, IND

**Keywords:** public health care, private sector, national digital health mission, indian healthcare system, global healthcare systems

## Abstract

The Indian healthcare system is a diverse and complex network of public and private sectors that provide a wide range of medical services to India's 1.4 billion inhabitants. Despite undergoing significant changes over the years, the system continues to face multiple challenges. These challenges include inadequate infrastructure, a shortage of healthcare professionals, urban-rural disparities, limited health insurance coverage, insufficient public healthcare funding, and a fragmented healthcare system. India is grappling with a growing burden of non-communicable diseases, which poses a significant challenge to its healthcare system.

The Indian government has initiated multiple programs to improve the healthcare system. The National Health Mission improves the availability of medical equipment and supplies. This also promotes community participation and engagement in healthcare decision-making and service delivery. The Ayushman Bharat scheme is a health insurance program that provides coverage of up to INR 5 lakhs per family per year for secondary and tertiary care hospitalization.

The Indian healthcare system is also witnessing multiple healthcare innovations, ranging from low-cost medical devices to innovative healthcare delivery models. The country's healthcare regulatory system is evolving to ensure patient safety, promote high-quality care, and control costs.

Furthermore, India has emerged as a leading destination for medical tourism due to the relatively low cost of medical procedures, the availability of skilled doctors, and advanced technology. Factors such as cost-effective treatment, advanced technology, a wide range of specialities, alternative medicine, English language proficiency, and ease of travel have contributed to India's growing medical tourism industry.

The Indian healthcare system has made significant progress in recent years. The positive transformation of the Indian healthcare system involves a range of changes and initiatives. Despite challenges, the continued investment in healthcare and innovation provides reasons to be optimistic about the future of healthcare in India.

## Editorial

The structure and organization of healthcare systems vary widely across different countries and regions. Some countries have a predominantly public healthcare system, where the government is responsible for providing healthcare services to the population. Other countries have a predominantly private healthcare system where healthcare services are provided by private hospitals. A well-functioning healthcare system provides high-quality healthcare services to the people, and it should be accessible, affordable, and sustainable over the long term [1].

The Indian healthcare system is a complex and diverse network made up of the public and private sectors, which offer a range of medical services and infrastructure to the 1.4 billion people living in India. It has undergone significant transformations over the years but still faces several challenges. The public sector includes primary, secondary, and tertiary care facilities managed by the central and state governments. Primary healthcare services are the individual's first point of contact and are provided through primary health centers, community health centers, and sub-centers. Secondary care focuses on acute and specialist services provided by district hospitals. Tertiary care refers to advanced medical services, including specialty and super-specialty services provided by medical colleges. The private sector consists of individual practitioners, nursing homes, clinics, and corporate hospitals [2].

The Indian healthcare system faces several challenges that impact its ability to deliver quality healthcare services to its large and diverse population [3]. Some of the key challenges are:

Inadequate infrastructure

India has a shortage of healthcare facilities, especially in rural areas, where the majority of the population resides. Many primary health centers and sub-centers lack essential infrastructure, medical equipment, and resources, making it difficult to provide even basic healthcare services to the population. The insufficient number of healthcare facilities, poorly maintained facilities, inadequate medical equipment and resources, and limited access to advanced healthcare services exacerbate the existing challenges in providing quality healthcare services to the population [3].

Shortage of healthcare professionals 

India has a significant shortage of healthcare professionals, including doctors, nurses, and paramedical staff. This is a critical challenge facing the Indian healthcare system, affecting the quality and accessibility of healthcare services across the country. The scarcity of trained medical staff has consequences like inadequate patient care. This is particularly evident in rural areas, where the majority of the population resides but has limited access to trained medical professionals. The limited capacity of medical and nursing schools to train healthcare professionals is a contributing factor to the shortage of skilled staff.

Urban-rural disparities

There is a marked disparity in the quality and accessibility of healthcare services between urban and rural areas. Urban areas tend to have better infrastructure, access to skilled professionals, and availability of specialized care, while rural areas often struggle with inadequate facilities and limited human resources.

Financial constraints and health insurance

The high out-of-pocket expenses for healthcare services can be a major burden for many Indians. Health insurance in India is not as widespread as in some other countries. This can lead to delayed or avoided treatments, causing further complications and health issues.

Insufficient public healthcare funding

The Indian government's expenditure on healthcare has historically been low compared to other countries, which contributes to the inadequacy of public healthcare facilities and the high reliance on private healthcare services, which may not be affordable for all citizens.

Fragmented healthcare system and inequity in access to care

The Indian healthcare system is characterized by a complex mix of public and private providers with varying degrees of quality and regulation. Socioeconomic disparities and regional differences in access to healthcare services result in unequal healthcare outcomes for different population groups, with poorer communities and those living in remote areas often facing greater challenges in accessing quality healthcare.

Growing burden of non-communicable and communicable diseases

Non-communicable diseases, such as diabetes, cardiovascular diseases, and cancer, have been on the rise in India, putting additional strain on the healthcare system. Despite progress in recent years, India still faces challenges in controlling communicable diseases like tuberculosis, malaria, and HIV/AIDS, which continue to pose significant public health risks.

The positive transformation of the Indian healthcare system is a multifaceted and ongoing process that involves many different changes and initiatives. The statistical data shows that the average life expectancy at birth in India has increased by approximately three years in the last ten years. The government has been working to improve the healthcare system through various initiatives to strengthen primary, secondary, and tertiary healthcare services. The Indian government spent two percent of India's gross domestic product (GDP) on healthcare in financial year 2022 and is forecast to reach over 2.5% of the GDP by 2025. In the financial year 2022, the government of India allocated approximately 860 billion Indian rupees to the Ministry of Health and Family Welfare in the Union Budget. The health tech sector in India secured private equity and venture capital investments worth nearly 1,740 million U.S. dollars in 2021. India's healthcare sector was worth about 280 billion U.S. dollars in 2020, and it was estimated to reach up to 372 billion dollars by 2022. The country's healthcare market had become one of the largest sectors in terms of revenue and employment, and the industry was growing rapidly [2].

Indians spend approximately 20 percent of their health spending as an out-of-pocket expenditure. In 2019, Indians spent around 55 percent of their total health spending as an out-of-pocket expenditure. This was at 74 percent in 2001, showing a gradual decrease in the share of healthcare expenses that people pay directly to the providers [2].

Some of the key elements of this positive transformation of India's healthcare system are the National Health Mission, Ayushman Bharat, and medical tourism.

The National Health Mission (NHM) was launched in 2013 and comprises the National Rural Health Mission (NRHM) and the National Urban Health Mission (NUHM). The NHM aims to strengthen primary healthcare infrastructure and services by upgrading existing facilities, building new ones, and improving the availability of medical equipment and supplies. This initiative also seeks to enhance human resources for health by training and recruiting more doctors, nurses, and paramedical staff, especially in rural areas. The NHM also aims to improve maternal, neonatal, and child health by expanding access to essential services such as antenatal care, skilled birth attendance, and immunization programs. Finally, it targets communicable and non-communicable diseases through targeted interventions and public health campaigns. The National Health Mission was allocated a budget of over 290 billion Indian rupees for the financial year 2024 [2].

Ayushman Bharat is another flagship healthcare initiative launched in 2018. This scheme provides financial protection and health coverage to India's vulnerable populations through Health and Wellness Centers (HWCs) and the Pradhan Mantri Jan Arogya Yojana (PMJAY). As of December 2022, there were about 117 thousand Ayushman Bharath Health and Wellness Centers (AB-HWCs) across India. AB-HWCs provide free essential medicine, diagnostic services, and teleconsultation. The HWCs aim to provide comprehensive primary healthcare services to rural and urban populations, including preventive, promotive, and curative care. The HWCs focus on maternal and child health, non-communicable diseases, communicable diseases, and palliative care while providing essential drugs and diagnostic services. The PMJAY is a health insurance scheme that provides coverage of up to INR 5 lakhs per family per year for secondary and tertiary care hospitalization. This initiative targets approximately 100 million economically disadvantaged families, covering around 500 million beneficiaries, and covers a range of medical procedures and treatments at empanelled hospitals. PMJAY aims to reduce out-of-pocket expenses and improve access to quality healthcare for India's poorest and most vulnerable populations. Over 217 thousand public health facilities were reported in India as of the financial year 2022. Over 1.4 billion services were performed by outpatient departments across India, a significant increase from the previous year's value of over 1.1 billion [2].

Digital healthcare

The shift towards digital healthcare in India is transforming the way healthcare services are delivered, particularly in remote areas. Telemedicine, digital health records, and mobile health apps are all being used to improve healthcare service quality and efficiency [4].

Non-communicable disease prevention and management

India is facing a growing burden of non-communicable diseases, but there are efforts underway to prevent and manage these diseases. This includes initiatives to promote healthy lifestyles, increase awareness of disease prevention, and provide specialized care and treatment for those with chronic conditions.

The penetration of health insurance across India stood at around 35 percent as of the financial year 2018. This was a slight increase compared to the previous year, when penetration levels were about 33 percent. In the financial year 2021, nearly 514 million people across India were covered under health insurance schemes, and the value of premiums for the government-sponsored health insurance schemes across India aggregated to around 43 billion Indian rupees [2].

Healthcare innovation and regulation

There are many examples of healthcare innovation happening in India, from low-cost medical devices to innovative healthcare delivery models. These innovations have the potential to improve healthcare outcomes and reduce costs in the long term. India's healthcare regulatory system is evolving to ensure patient safety, promote high-quality care, and control costs. The government is taking steps to streamline the regulatory system and ensure that healthcare providers adhere to high standards of care [5].

The private healthcare sector in India plays a vital role in achieving universal health coverage, as recognized by the government. India offers healthcare services at comparatively low costs, attracting international patients seeking quality treatment at affordable prices. The private healthcare sector has made significant advancements in infrastructure, technology, specialized services, and healthcare access. Private healthcare providers have invested in modern hospitals, clinics, and diagnostic centers equipped with advanced medical technology. They have embraced digital innovations such as electronic medical records, telemedicine, health apps, and remote monitoring systems to improve patient care. Increased health insurance coverage has facilitated access to private healthcare services, with insurance companies collaborating with private hospitals and clinics. The government has encouraged public-private partnerships to enhance healthcare access and infrastructure, particularly in underserved areas. Collaborative efforts between the public and private sectors, along with targeted interventions, can help bridge gaps and create a more inclusive healthcare system.

Medical tourism

India has become a popular destination and thrived due to the availability of advanced treatments at relatively lower costs, the availability of skilled doctors and advanced technology in private hospitals contributing to foreign exchange earnings, and a positive reputation. India has emerged as a popular destination for medical tourism in recent years, attracting patients from around the world. The factors contributing to India's growing medical tourism industry include cost-effective treatment, skilled medical professionals, advanced technology, a wide range of specialties, alternative medicine, English language proficiency, and ease of travel.

Despite the challenges, the Indian healthcare system has made significant positive progress in recent years, particularly in terms of expanding access to healthcare services and improving health outcomes. These government initiatives, programs, and policies address the various challenges faced by the Indian healthcare system and improve access to quality healthcare services for all citizens. The positive transformation of India's healthcare system is ongoing and involves a range of changes and initiatives. While there are still significant challenges to overcome, such as healthcare access disparities and the burden of disease, the continued investment in healthcare and innovation in the sector are reasons to be optimistic about the future of healthcare in India. However, sustained efforts and investments are required to ensure that the benefits of these initiatives reach the intended beneficiaries and lead to lasting improvements in health outcomes.
